# Reliable calibration by nonlinear standard addition method in the presence of additive interference effects

**DOI:** 10.1007/s00706-018-2203-1

**Published:** 2018-08-07

**Authors:** Marcin Wieczorek, Marek Dębosz, Paweł Świt, Aleksandra Piech, Joanna Kasperek, Paweł Kościelniak

**Affiliations:** Department of Analytical Chemistry, Faculty of Chemistry, Jagiellonian University in Kraków, Kraków, Poland

**Keywords:** Spectrophotometry, Calibration methods, Interference effects, Nonlinear calibration dependence

## Abstract

**Abstract:**

The possibility of adapting the Standard Addition Method (SAM) to calibration in very difficult analytical conditions, namely when there is a need to determine an analyte with the use of nonlinear calibration graph and in the presence of matrix components causing additive interference effect, is investigated. To this aim the SAM in the common version and the Chemical H-point Standard Addition Method (C-HPSAM) realized by the flow injection technique were applied. Specifically, a flow manifold was used for construction of a set of nonlinear calibration graphs in different chemical conditions. As the graphs were intersected indicating both the additive interference effect and the analytical result free of this effect, the analyte concentration in the sample was able to be obtained with improved accuracy. The applicability of this approach was verified on the example of spectrophotometric determination of paracetamol in pharmaceuticals and of total acidity in wines. The C-HPSAM method enabled complete compensation of the additive effect and obtaining analytical results at a relative error not exceeding 6.0%.

**Graphical abstract:**

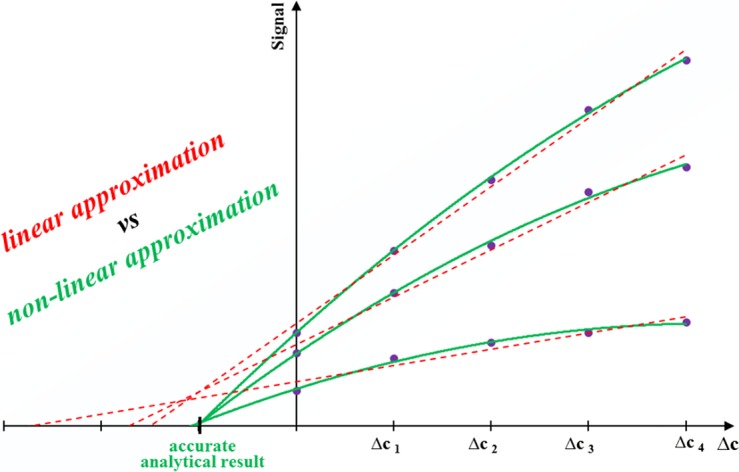

## Introduction

The analysis of the samples with unknown, complex matrices still causes a lot of problems. The influence of the matrix components on the analytical signal, i.e., the interference effect, can lead to the analyte determination with serious systematic error. Because of this, the chemists must pay particular attention to the elimination of this effect at either the sample pre-treatment or the calibration stage.

One of the well-known calibration approaches allowing for the interferences to be minimized is the Standard Addition Method (SAM). In the basic version it consists in addition of known, increasing amounts of an analyte to the same portions of the sample, then dilution of all solutions to the same volume and measurement of analytical signal for the whole concentration of the analyte in the prepared solutions. The analyte concentration is calculated from the calibration function extrapolated to zero value of the analytical signal. Due to the presence of all components (including possible interferents) in the calibration solutions the interference effect can be effectively compensated for independently of kind and concentration of interferents in the sample.

Although SAM has important advantages, it is used only occasionally in fact. One of the reasons is that the set of calibration solutions have to be prepared for each examined sample separately. However, the calibration procedure can be easily automated and accelerated using special techniques [1, 2], including the flow ones [3, 4]. More serious problem is that the extrapolation process is a source of greater random errors than the interpolative way typical for the calibration curve method (CCM) conventionally used in analytical practice [5]. Special caution against employment of SAM is recommended if the nonlinear calibration function is fitted to the measurement points. In this case, the general opinion is that the reliability of SAM in terms of precision worsens distinctly [5, 6]. It is also suggested to avoid the nonlinearity for the reason of accuracy, particularly if the calibration curve is suspected to be described by different functions in the parts experimentally established and extrapolated [7]. The conclusion of the investigations in this field is best reflected by Welz’s point of view: ‘‘…the analyte addition technique can be applied without limitations… only within the linear range of the analytical curve” [6].

However, whether we like it or not, it is necessary to perform analyses in the nonlinear calibration range quite often, especially when the nonlinearity is natural (caused by, e.g., instrumental reasons) and it can be avoided by dilution of the calibration solutions. It was shown that if the experimental points are distributed even slightly nonlinearly (almost unnoticeably) but systematically, one should not force to fit them in SAM by linear function as the analytical results obtained in extrapolative way can be seriously erroneous [8]. On the other hand, it was mathematically and experimentally proved in the same work [8] that if the distribution of points is only slightly curved, the nonlinear calibration by SAM is not only allowed, but even favourable in comparison with linear calibration in terms of both precision and accuracy of the final results.

The undisputed limitation of SAM (similarly to CCM) is that the method is able to reduce interference effect only when it is proportional (multiplicative) and not constant (additive) in relation to the analyte concentration. It results from the composition of the calibration solutions, which contain the constant concentration of interferents in the presence of increased concentrations of the analyte. This problem can be overcome using SAM in the form of the H-point Standard Addition Method (HPSAM) [9]. In accordance to this approach, the SAM procedure is performed under two different strictly defined conditions (usually wavelengths) selected so that the signal measured for interferents is constant while the signal measured for an analyte is as much as possible different. As a consequence, the calibration lines obtained in both conditions are intersected in a point (H-point) indicating, both the constant value of the signal corresponding to the additive interference effect and the analytical result free of this effect.

The disadvantage of the HPSAM is that its prerequisite is very restrictive, hence in most cases the method requires knowledge on what sample components play a role of interferents and which of them can cause the additive effect. To increase the applicability and reliability of the method its chemical version has been recently proposed [10]. It consists in realization of the HPSAM procedure in two (or even more) chemical and not instrumental conditions. In addition, it has been recommended to automate the procedure with the use of dedicated flow manifolds.

Independently of the version and procedural modifications HPSAM was applied so far in the linear mode, i.e., the SAM calibration lines serving for estimation of the additive interferences and the analytical result were developed in linear signal vs. concentration range. The present paper describes, for the first time, the reliability and effectivity of HPSAM applied in nonlinear mode. Based on our experience, the chemical version realized by flow injection technique was exploited. The method was tested on the examples of the spectrophotometric determinations of paracetamol in pharmaceuticals and of total acidity in wines.

## Results and discussion

### Determination of paracetamol

**Fig. 1:**
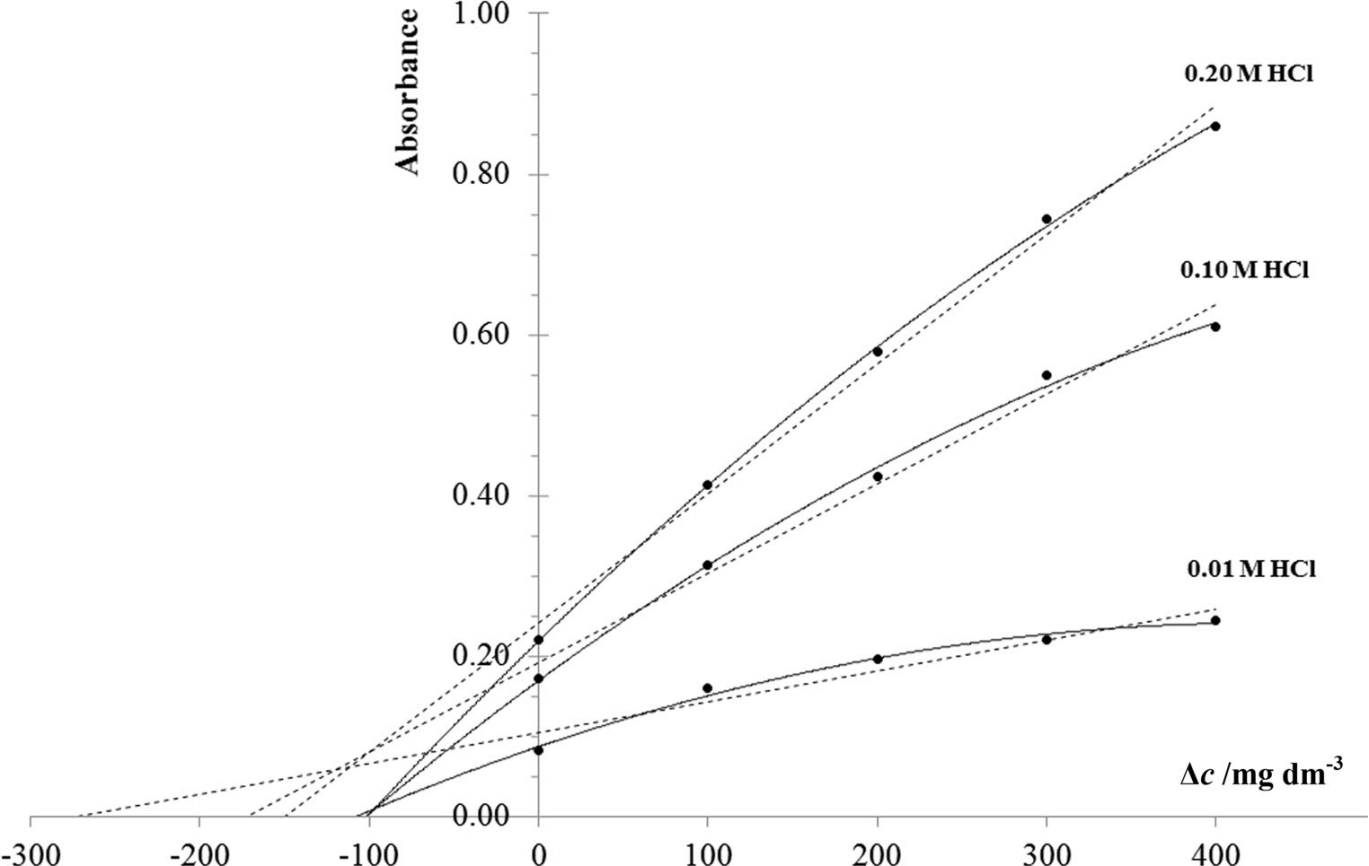
Calibration graphs obtained for a synthetic sample of paracetamol (100 mg dm^−3^)

Figure 1 shows the SAM calibration curves obtained for a synthetic sample (containing 100 mg dm^−3^ of paracetamol only) by fitting linear and nonlinear (polynomial) functions to the same experimental points. Each point on the SAM calibration curve is the average of three repetitions of the measurement. The final results for the SAM calibration procedure, either linear or nonlinear, were obtained as the average of three intercept points with abscissa axis. Whereas the final results for the C-HPSAM were obtained as the intersection of three, either linear or nonlinear, SAM calibration curves.

Normally, for the synthetic sample without the interferents, the calibration curves should intersect in one point on the abscissa axis. This can be seen in Fig. 1 for the case of nonlinear approximation that surpluses the linear fit which does not follow the aforementioned property. However, the C-HPSAM seems to be more appropriate calibration procedure since it is more robust to the chosen kind of approximation.

Table 1 collects results obtained with the use of two types of calibration methods: Standard Addition Method (SAM) with the linear and polynomial approximations and Chemical H-point Standard Addition Method (C-HPSAM) with nonlinear fit. The concentration values are given along with *T* test confidence interval (with 5% significance level). The use of SAM with linear approximation is associated with unacceptable levels of relative errors, while the use of either nonlinear SAM or nonlinear HPSAM results in more accurate values of concentrations. However, in the case of synthetic sample with fruit colorant, the implementation of SAM with polynomial approximations does not seem to be useful since the analytical result is still far from expected concentration. This might be explained by the fact that the SAM does not compensate the influence of additive (non-specific) interference effect on the final result. Such compensation might be achieved by the HPSAM (in that case by chemical version of HPSAM), that allows for the compensation of both kinds of interference effects—multiplicative (specific) and additive (unspecific). That is why for the synthetic sample with colorant, where the SAM approach failed, the C-HPSAM delivers the final result with small relative error. When it comes to the real samples, the tendency is similar, when comparing the calibration methods. For the analysed samples, (not associated with the additive interference effect), the use of C-HPSAM helps obtaining more accurate results than SAM. For the real samples, the C-HPSAM based on nonlinear approximation seems to be the obvious choice since the results for this calibration method deliver the lowest values of the relative error.

**Table 1:** Results (concentration with confidence interval) obtained in paracetamol determination; RE—relative error

Sample	Expected conc. *c*_0/_mg dm^−3^	Linear approximation		Nonlinear approximation			
		SAM		SAM		C-HPSAM	
		Conc./mg dm^−3^	RE/%	Conc./mg dm^−3^	RE/%	Conc./mg dm^−3^	RE/%
Synthetic	100.00	200.07 ± 114.56	100.1	103.88 ± 6.48	3.9	96.88 ± 15.72	− 3.1
Synthetic with fruit colorant	100.00	615.84 ± 646.80	515.8	344.55 ± 193.15	244.6	97.89 ± 19.86	− 2.1
Febrisan^a^	75	121.59 ± 15.52	62.1	82.61 ± 2.83	10.2	79.10 ± 19.36	5.5
Vicks^a^	50	82.29 ± 1.85	64.6	51.91 ± 14.48	3.8	50.33 ± 17.11	0.7
Theraflu^a^	65	105.72 ± 2.13	62.6	67.24 ± 15.66	3.4	65.79 ± 8.85	1.2

^a^The expected concentration is based on the declaration of the pharmaceutical manufactures

### Determination of total acidity

Table 2 presents the results obtained during the determination of total acidity in wine samples using SAM and C-HPSAM method in the nonlinear mode. The results obtained by SAM are accurate only for two white wines samples (Sophia Trakia white and Carlo Rossi white) and are not accurate for other samples, although they already take into account the nonlinear nature of the calibration relationship. For rose wines, the results obtained by SAM are lower than expected, which may indicate the occurrence of a negative additive interference effect. The color of the wine causes that the absolute value of the negative peak is lower causing a negative systematic error of the determination by SAM. Only the use of C-HPSAM makes it possible to compensate the additive interference effect and obtaining accurate results for both white and rose wines.

**Table 2:** Results obtained in total acidity determination; RE—relative error

Sample	Expected value *c*_0_/mmol dm^−3^	Nonlinear SAM		Nonlinear C-HPSAM	
		/mmol dm^−3^	RE/%	/mmol dm^−3^	RE/%
Portada white	37.76 ± 1.89	33.14 ± 2.91	− 12.2	36.54 ± 4.25	− 3.2
Imiglykos white	35.67 ± 0.87	31.12 ± 2.64	− 12.7	37.46 ± 3.74	5.0
Sophia Trakia white	32.71 ± 1.32	33.87 ± 3.45	3.6	33.49 ± 3.32	2.4
Carlo Rossi white	44.96 ± 2.49	47.22 ± 3.46	5.0	43.92 ± 2.08	− 2.3
Bordeaux rose	48.49 ± 1.49	38.25 ± 3.52	− 21.1	49.08 ± 4.45	1.2
Carlo Rossi rose	35.56 ± 0.82	29.87 ± 3.55	− 19.0	37.36 ± 1.52	5.1
Fresco rose	50.52 ± 4.44	43.44 ± 2.85	− 14.0	47.69 ± 2.23	− 5.6

## Conclusions

As it has been shown, the accuracy of a result in the SAM method depends on correct reflection of the calibration relationship. The use of a linear graph in the case of even a slight nonlinearity of the calibration relationship leads to inaccurate results. However, contrary to the popular belief, the SAM method can be used in the case of a nonlinear calibration dependence provided that the graph is approximated with an appropriate nonlinear function, which results in a significant improvement in accuracy of the obtained results. However, in some cases, despite the use of a nonlinear calibration graph, results obtained by SAM may still be subject to a systematic error, which is caused by the additive interference effect. As it has been proven, in this case the use of the SAM method in the C-HPSAM version in nonlinear mode allows for compensation of both multiplicative and additive effects and obtaining results with very good accuracy.

## Experimental

### Reagents and solutions

The paracetamol stock solution 5 g dm^−3^ was prepared by dissolving 0.5 g of paracetamol (Acetaminophen, Sigma-Aldrich, Germany) in 100 cm^3^ of water. The solution served for preparation of the synthetic sample (of 100 mg dm^−3^) and a set of calibration solutions (the sample dozed with standards). The solutions of hydrochloric acid of 0.01, 0.1, and 0.2 mol dm^−3^ were prepared by appropriate dilution of 37% HCl (Merck, Germany) with water. The NaNO_3_ solution of 0.14 mol dm^−3^ was prepared by dissolving 4.83 g of NaNO_3_ (POCH, Poland) in 500 cm^3^ of water. The NaOH solution of 0.1 mol dm^−3^ was prepared by dissolving 4 g of NaOH (POCH, Poland) in 1 dm^3^ of water. The food dye working solution of 1.2 g dm^−3^ was prepared by dissolving 60 mg of E110 dye (Hokus, Poland) in 1.25 cm^3^ of EtOH and 48.75 cm^3^ of 0.1 mol dm^−3^ HCl.

The tartaric acid stock solution of 0.2 mol dm^−3^ was prepared by dissolving 3 g of C_4_H_6_O_6_ (POCH, Poland) in 100 cm^3^ of water. The solution served for preparation of the synthetic sample (of 0.020 mol dm^−3^) and a set of calibration solutions. The phosphate buffer of 0.2 mol dm^−3^ was prepared by mixing 947 cm^3^ of 0.2 mol dm^−3^ Na_2_HPO_4_ (Lach-Ner, Czech Republic) and 53 cm^3^ of 0.2 mol dm^−3^ KH_2_PO_4_ (POCh, Poland). The solution of 3.3 mmol dm^−3^ of bromothymol blue (used as indicator) was prepared by dissolving 0.206 g of solid bromothymol blue (The British Drug Houses, UK) in 5 cm^3^ of 96% ethanol (POCH, Gliwice). All chemicals were of analytical grade and the ultrapure water (18.2 MΩ cm) from HLP 5 system (Hydrolab, Poland) was used throughout the work.

### Samples

Paracetamol was determined in two synthetic samples containing 100 mg dm^−3^ of the analyte alone and with the addition of 24 mg dm^−3^ of dye. Three real pharmaceutical samples were also prepared: Theraflu ExtraGrip (GlaxoSmithKline Consumer Healthcare, Poland), Vicks SymptoMed Complete (Teva Pharmaceuticals, Poland) and Febrisan (Takeda, Poland). Each sample was dissolved in 100 cm^3^ of water in an ultrasonic bath for 10 min. The mass of analyzed samples were 14.85, 4.36, and 5.00 g, respectively. Obtained solutions were filtrated with cellulose filters and degassed in an ultrasonic bath for 10 min. The Febrisan sample was diluted one hundred times in distilled water whereas the Vicks Complete and Theraflu ExtraGrip samples were diluted fifty times in pure water. Based on the declaration of the pharmaceutical manufactures the paracetamol concentrations in the above samples prepared in described way were expected to be 75, 50, and 65 mg dm^−3^, respectively.

Total acidity was determined in the wine samples: Sophia Trakia white (Vinprom Byala, Bulgaria), Bordeaux rose (Producta, France); Fresco rose (Ambra S.A., Poland), Carlo Rossi rose (Carlo Rossi Vineyards, USA), Portada white (Jose Neiva Correia, Portugal), Carlo Rossi white (Carlo Rossi Vineyards, USA), Imiglykos white (Mediterra, Greece). The expected acidity concentrations in the sample were obtained by potentiometric titration as the reference method. Prior to titration the samples were pre-treated by adding 25 drops of 35% H_2_O_2_ (Merck, Germany) to 50 cm^3^ of the sample and stirring for 3 min in closed flask subjected to vacuum for removal of the carbon dioxide. Each assay was repeated three times and the result was determined based on the Hahn method.

### Instrumentation

**Fig. 2:**
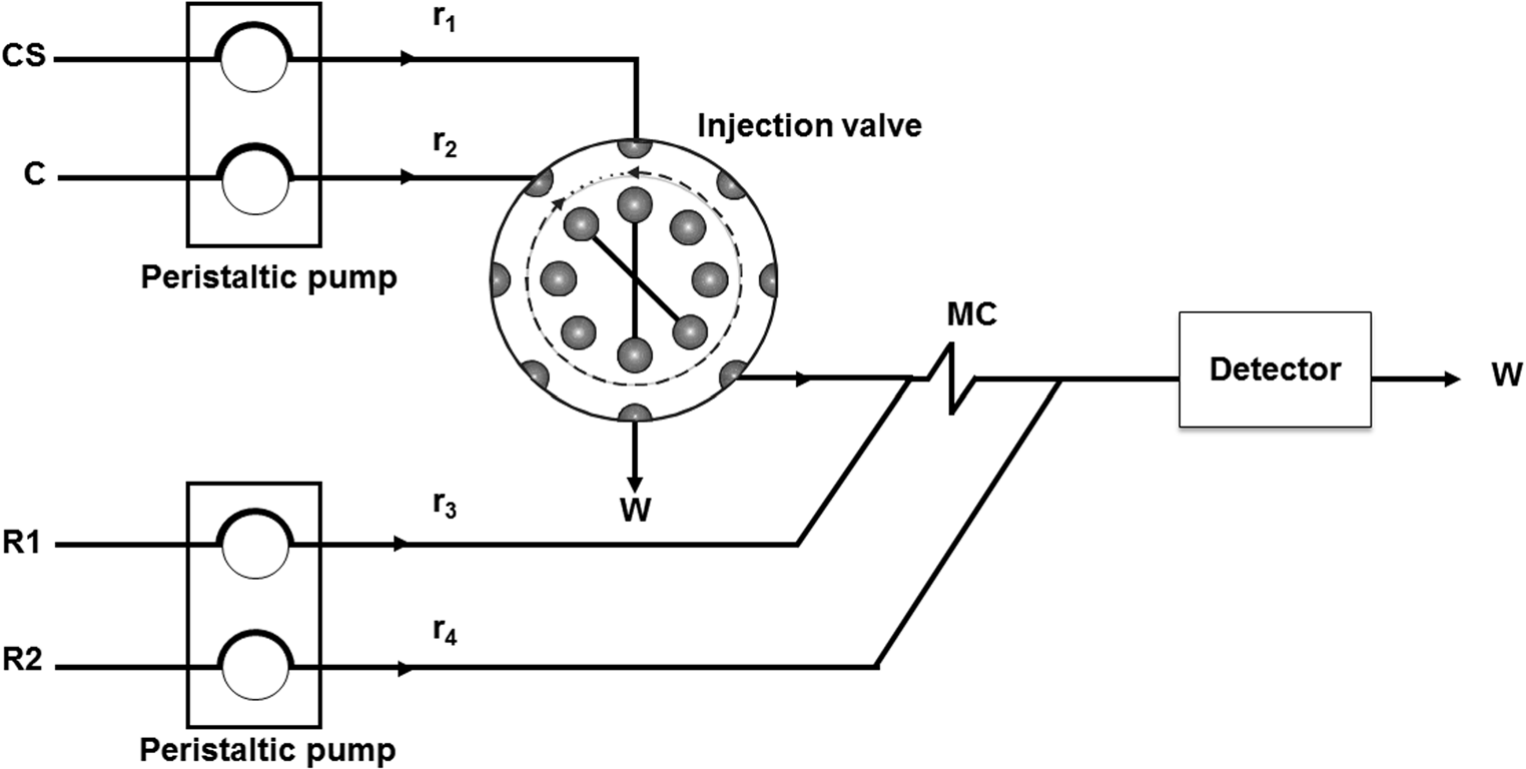
Scheme of the manifold used throughout the experiments: *CS* calibration solution, *C* carrier, *R1, R2* reagents, *MC* mixing coil, *detector* spectrophotometer, *W* waste

The flow injection manifold used for the determination of both paracetamol and acidity is shown in Fig. 2. It was equipped with two peristaltic pumps (Minipuls 3, Gilson, France) and an injection valve (Perkin Elmer, USA) operated by a homemade control system. Lambda 25 spectrometer (PerkinElmer, USA) equipped with a glass flow cell with length of 10 mm (Hellma Gmb & Co., Germany) was used as the detector.

A 16-channel controller UVCTR-16 (KSP Elektronika Laboratoryjna, Poland) with Valve and Pump Controller software (KSP Electronics Laboratory, Poland) was utilized to control pumps and the valve.

The potentiometric titration of the wine samples was performed with the use of and potentiometer CPI-501 (Elmetron, Poland) equipped with the pH electrode ERH-11S (Elmetron, Poland).

In the case of the paracetamol determination the following parameters of the flow manifold were found as optimum (see Fig. 2): injection loop volume: 70 mm^3^, flow rates: *r*_1_ = 2.0 cm^3^ min^−1^: *r*_2_ = 2.0 cm^3^ min^−1^: *r*_3_ = 2.0 cm^3^ min^−1^: *r*_4_ = 2.0 cm^3^ min^−1^, mixing coil length = 100 cm. Acidity was determined in the following optimum conditions: injection loop volume: 100 mm^3^, flow rates: *r*_1_ = 3.4 cm^3^ min^−1^: *r*_2_ = 3.4 cm^3^ min^−1^: *r*_3_ = 3.4 cm^3^ min^−1^: *r*_4_ = 0 cm^3^ min^−1^, mixing coil length = 100 cm.

### Procedures

For the determination of paracetamol the analytical method reported in [11] was adapted to the C-HPSAM procedure. The employed method was based on nitrification of paracetamol in reaction with sodium nitrate in acidic environment of hydrochloric acid of different concentrations. The obtained derivative species reacted further with sodium hydroxide to convert it into a more stable compound for which absorbance was measured at 430 nm. Each sample was dosed with the standard solutions in concentrations 0, 100, 200, 300, and 400 mg dm^−3^. All of solutions were prepared manually. The calibration solutions (CS) were successively introduced to the flow manifold (see Fig. 2) and injected to the HCl solution as the carrier stream (C). In the stream of sodium nitrate(III) solution (R1) a nitroso derivative of the analyte was formed, which was stabilized with the sodium hydroxide solution (R2). The yellow reaction product was recorded at 430 nm. The signals were measured in the peak height mode. All calibration solutions were injected to HCl of different concentrations, i.e., 0.01, 0.10, and 0.20 mg dm^−3^, to differentiate the reaction conditions and to obtain three calibration graphs of different sensitivity. The entire procedure was repeated three times in the same instrumental conditions and the mean values were taken for further calculations.

In the case of the acidity determination a set of calibration solutions (CS) containing a sample dosed with 0, 20, 40, 60, and 80 mmol dm^−3^ of the analyte, prepared separately, was successively injected to water as the carrier stream and then merged with the bromothymol blue solution (R1). The signals were recorded at 615 nm as negative peaks resulting from discoloration of the indicator. Analytical signal was measured as the difference between the baseline signal and the minimum signal indicated by the flow peak. Each determination was repeated three times in the same instrumental conditions.
